# Supplementary material for: Healthcare practitioner experiences and willingness to prescribe pre-exposure prophylaxis in the US
Source: PLoS One. 2020 Sep 3;15(9):e0238375. doi: 10.1371/journal.pone.0238375 (PMC7470257; doi:10.1371/journal.pone.0238375)
Supplement: S1 File — This file comprises of the survey distributed at IAS-USA CME courses in five locations across the US (San Francisco, CA, Los Angeles, CA, New York, NY, Washington, DC, and Chicago, IL). (DOCX) [file pone.0238375.s001.docx]

**S1 File: Research survey conducted at IAS-USA Continuing Medical Education (CME) practitioner course**

The purpose of this survey is to evaluate whether providers differ in their experience and perceptions around prescribing indications and the efficacy of utilization of PrEP. This survey examines practitioner perceptions of prescribing PrEP across different indications compared to other methods in addition to gauging one’s experiences with this medication. Participation in the survey is voluntary and will take approximately 5-7 minutes to complete. Please contact Ashley Leech at 917-816-1055 or by email at [ashleyl@bu.edu](mailto:ashleyl@bu.edu) with any questions about the research.

1. Age: _________________________
2. Gender:  Male  Female  Other: ____________________
3. Which ethnic and racial group(s) do you identify with?

Hispanic/Latino  White

American Indian or Alaska Native  Black or African American

Native Hawaiian or other Pacific Islander  Asian

Other, specify: ____________________

1. In what **State** do you practice? ________________________________________
2. Please state your qualification/licensure:

Doctor of Medicine (MD) /  Physician Assistant (PA)

Doctor of Osteopathic Medicine (DO)  PhD

Nurse Practitioner (NP)  Other (please specify): ________________________

Registered Nurse (RN)

1. How long have you been practicing independently?

In training  11-20 years  5-10 years

<5 years  21+ years

1. What is your specialty area?

Infectious diseases  Internal medicine

Family medicine  Obstetrics/gynecology

Pediatrics  Other (please specify): _________________________

1. Have you prescribed PrEP?

Yes  No

8a. If yes, what has been the **primary** population to which you have prescribed?

Anticipated men who have sex with men (MSM) exposure

Anticipated intravenous drug-user (IDU) exposure

Anticipated at-risk heterosexual exposure (not trying to conceive)

Anticipated conception exposure

Other (please specify): _________________________

1. How often in the course of routine care do you typically ask your patients their partners’ HIV sero-status?

Never  Rarely  Sometimes  Most of the time  Always

1. How often in the course of routine care do you typically ask your **HIV+ male** patients their partners’ HIV sero-status?

Never  Rarely  Sometimes  Most of the time  Always

1. How often in the course of routine care do you typically ask your **HIV+ female** patients their partners’ HIV sero-status?

Never  Rarely  Sometimes  Most of the time  Always

*Please state whether you “strongly agree,” “agree,” are “neutral,” “disagree,” or “strongly disagree” with the following:*

1. If cost and access were not barriers, I would refer all of my HIV-infected patients in sero-discordant relationships who wish to conceive for assisted reproductive technologies.

Strongly Agree  Agree  Neutral  Disagree  Strongly Disagree

1. I am comfortable counseling my HIV-infected patients on safe options for conception/pregnancy.

Strongly Agree  Agree  Neutral  Disagree  Strongly Disagree

*The following case vignettes are followed by specific clinical options, none of which can be considered either correct or incorrect.* ***Please choose one option as the best first step*** *in your opinion.*

1. A 30-year-old male who is HIV-negative and has sex with men with an unremarkable PMH is sexually active with his HIV-positive husband. The (+) partner is on cART but having challenges reaching an undetectable HIV viral load. What do you recommend as a first step?

Use condoms

Recommend initiating PrEP for HIV-negative partner

Use both condoms and initiate PrEP for HIV-negative partner

Other (please specify): ______________________________________________________________

1. A 30-year old female who is HIV-negative with an unremarkable PMH is hoping to conceive with her HIV-positive partner. The (+) partner is on cART with an undetectable HIV viral load. What do you recommend as a first step?

Do not conceive

Recommend initiating PrEP for HIV-negative woman

Refer couple for Assisted Reproductive Technology services

Other (please specify): ______________________________________________________________

1. A 30-year old male who is HIV-negative with a history of injection drug use reports sharing needles with injection partners of unknown sero-status. What do you recommend as a first step?

Refer to harm reduction program only, such as safe needle exchange

Refer to substance abuse treatment and harm reduction program such as safe needle exchange if exists

Recommend initiating PrEP

Other (please specify): ______________________________________________________________

1. What barriers, if any, do you **foresee** OR have **encountered** in implementing PrEP for **conception** purposes?

__________________________________________________________________________________________________________________________________
