# Supplementary material for: Healthcare practitioner experiences and willingness to prescribe pre-exposure prophylaxis in the US
Source: PLoS One. 2020 Sep 3;15(9):e0238375. doi: 10.1371/journal.pone.0238375 (PMC7470257; doi:10.1371/journal.pone.0238375)
Supplement: S3 File — This file encompasses the relevant statistical output for the multivariable logistic regression analyses presented in the Results section. (DOCX) [file pone.0238375.s003.docx]

**S3 File: Statistical Output**

**PrEP prescribing patterns**

| **S3 Table 1.** Logistic Regression [Unadjusted Analysis]  Outcome = Experience prescribing PrEP (yes/no) | | | | |  |  |
| --- | --- | --- | --- | --- | --- | --- |
| N=515 (4 observations were deleted due to missing values for the outcome variable)  Global null hypothesis: BETA=0; likelihood ratio Chi-Square= 5.7857; Pr>ChiSq= 0.1225 | | | | | | |
| Parameter | **Estimate** | **Standard Error** | **Wald Chi-Square** | **Pr > ChiSq** | **Odds ratio** | **95% CI** |
| Intercept | 0.113 | 0.143 | 0.620 | 0.431 |  |  |
| Internal Medicine | 0.362 | 0.229 | 2.501 | 0.114 | 1.436 | 0.917-2.249 |
| Family Medicine | -0.113 | 0.222 | 0.259 | 0.61 | 0.893 | 0.578-1.379 |
| Other | -0.327 | 0.327 | 0.999 | 0.317 | 0.721 | 0.380-1.368 |
| Reference: Infectious Disease | | | | | | |

| **S3 Table 2.** Logistic Regression [Adjusted Analysis]  Outcome = Experience prescribing PrEP (yes/no) | | | | | | | |
| --- | --- | --- | --- | --- | --- | --- | --- |
| N=494 (21 observations were deleted due to missing values for the explanatory variables)  Global null hypothesis: BETA=0; likelihood ratio Chi-Square= 25.5150; Pr>ChiSq= 0.0025 | | | | | | | |
| Parameter | **Estimate** | **Standard Error** | **Wald Chi-Square** | **Pr > ChiSq** | **Odds ratio** | **95% CI** | |
| Intercept | 3.006 | 0.849 | 12.521 | <.001 |  |  | |
| Internal Medicine | 0.475 | 0.245 | 3.761 | 0.052 | 1.609 | 0.995-2.601 | |
| Family Medicine | -0.183 | 0.234 | 0.610 | 0.435 | 0.833 | 0.526-1.318 | |
| Other | -0.283 | 0.340 | 0.690 | 0.406 | 0.754 | 0.387-1.468 | |
| Reference: Infectious Disease | | | | |  |  | |
| Age | -0.043 | 0.013 | 10.255 | 0.001 | 0.958 | 0.933-0.983 | |
| Female | -0.556 | 0.195 | 8.101 | 0.004 | 0.574 | 0.391-0.841 | |
| In training | -1.211 | 0.526 | 5.296 | 0.021 | 0.298 | 0.106-0.836 | |
| 11–20 years’ experience | -0.440 | 0.279 | 2.488 | 0.115 | 0.644 | 0.373-1.113 | |
| 5–10 years’ experience | -0.624 | 0.369 | 2.870 | 0.090 | 0.536 | 0.260-1.103 | |
| < 5 years’ experience | -0.730 | 0.413 | 3.126 | 0.077 | 0.482 | 0.214-1.082 | |
| Reference: 20+ years’ experience | | | | |  | |  |

**Willingness to prescribe PrEP as a “best first step” to different risk categories**

| **S3 Table 3**. Logistic Regression [Unadjusted Analysis]  **Outcome = Safer conception**; N=502 | | | | | | |
| --- | --- | --- | --- | --- | --- | --- |
| Parameter | **Estimate** | **Standard Error** | **Wald Chi-Square** | **Pr > ChiSq** | **Odds ratio** | **95% CI** |
| Intercept | 0.505 | 0.289 | 3.066 | 0.080 |  |  |
| MSM | -0.343 | 0.309 | 1.238 | 0.266 | 0.709 | 0.388-1.299 |
| PWID | 0.767 | 0.204 | 14.182 | <.001 | 2.154 | 1.445-3.211 |

*Safer conception is the outcome variable*

| **S3 Table 4.** Logistic Regression [Adjusted Analysis]  **Outcome = Safer conception;** N=502  Global null hypothesis: BETA=0; likelihood ratio Chi-Square= 17.0278; Pr>ChiSq=0.0044 | | | | | | |
| --- | --- | --- | --- | --- | --- | --- |
| Parameter | **Estimate** | **Standard Error** | **Wald Chi-Square** | **Pr > ChiSq** | **Odds ratio** | **95% CI** |
| Intercept | 0.371 | 0.312 | 1.415 | 0.234 |  |  |
| MSM | -0.288 | 0.312 | 0.853 | 0.356 | 0.750 | 0.406-1.382 |
| PWID | 0.736 | 0.206 | 12.787 | <0.001 | 2.088 | 1.395-3.127 |
| Internal Medicine | 0.115 | 0.238 | 0.235 | 0.628 | 1.122 | 0.704-1.790 |
| Family Medicine | 0.292 | 0.238 | 1.507 | 0.220 | 1.339 | 0.840-2.135 |
| Other specialty | -0.120 | 0.346 | 0.121 | 0.727 | 0.886 | 0.450-1.746 |
| Reference: Infectious Disease | | | | | | |
| Safer conception is the outcome variable | | | | | | |

| **S3 Table 5.** Logistic Regression [Unadjusted Analysis]  **Outcome = PWID;** N=502 | | | | | | |
| --- | --- | --- | --- | --- | --- | --- |
| Parameter | **Estimate** | **Standard Error** | **Wald Chi-Square** | **Pr > ChiSq** | **Odds ratio** | **95% CI** |
| Intercept | -2.6006 | 0.4631 | 31.5350 | <.0001 |  |  |
| MSM | 1.6394 | 0.4475 | 13.4225 | 0.0002 | 5.152 | 2.143-12.384 |
| Conception | 0.7674 | 0.2037 | 14.1900 | 0.0002 | 2.154 | 1.445-3.211 |
| PWID is the outcome variable | | | | | | |

| **S3 Table 6.** Logistic Regression (Adjusted Analysis]  **Outcome = PWID;** N=502  Global null hypothesis: BETA=0; likelihood ratio Chi-Square= 43.6147; Pr>ChiSq <0.0001 | | | | | | |
| --- | --- | --- | --- | --- | --- | --- |
| Parameter | **Estimate** | **Standard Error** | **Wald Chi-Square** | **Pr > ChiSq** | **Odds ratio** | **95% CI** |
| Intercept | -3.0798 | 0.4969 | 38.4211 | <.0001 |  |  |
| MSM | 1.7162 | 0.4547 | 14.2453 | 0.0002 | 5.563 | 2.282 – 13.564 |
| Conception | 0.7515 | 0.2064 | 13.2573 | 0.0003 | 2.120 | 1.415 – 3.177 |
| Internal Medicine | 0.6642 | 0.2480 | 7.1752 | 0.0074 | 1.943 | 1.195 – 3.159 |
| Family Medicine | 0.7187 | 0.2477 | 8.4176 | 0.0037 | 2.052 | 1.263 – 3.334 |
| Other specialty | 0.4354 | 0.3622 | 1.4445 | 0.2294 | 1.546 | 0.760 – 3.144 |
| Reference: Infectious Disease | | | | | | |
| PWID is the outcome variable | | | | | | |

| **S3 Table 7.** Logistic Regression [Unadjusted Analysis]  **Outcome = MSM;** N=502 | | | | | | |
| --- | --- | --- | --- | --- | --- | --- |
| Parameter | **Estimate** | **Standard Error** | **Wald Chi-Square** | **Pr > ChiSq** | **Odds ratio** | **95% CI** |
| Intercept | 1.9738 | 0.2463 | 64.2324 | <.0001 |  |  |
| Conception | -0.3433 | 0.3086 | 1.2378 | 0.2659 | 0.709 | 0.387-1.299 |
| PWID | 1.6394 | 0.4475 | 13.4224 | 0.0002 | 5.152 | 2.143-12.384 |
| MSM is the outcome variable  *Included the unadjusted analysis here as reference, as we indicated “vice versa” in the text in regard to relationships between “safer conception & MSM,” “safer conception & PWID,” and “PWID & MSM” – therefore, only S3 tables 3-6 are necessary to include | | | | | | |
